# Mental Cure

**Published:** 1886-05

**Authors:** 


### MENTAL CURE.

In our January number, we stated some cases of mind cure from which we concluded that they did not always arise from faith, for people were sometimes cured who had no faith whatever, that the cure would be accomplished. We stated cures that we, ourselves knew about, when cures were made when the person cured did not know that there was an attempt to cure them, nor did the person who cured them or through whom they were cured, know that such a thing was to be done, How it was done we did not state for we did not know; but, that it was done we do positively know. We clip the following from the Boston *Transcript*, which gives some account of a work on that subject published by Colby & Rich of that city.

Dr. Warren F. Evans's "Mental Cure," illustrating the influence of the mind on the body, both in health and disease, and suggesting the psychological method of treatment, has passed to a seventh edition. Dr. Evans is unquestionably the ablest of the multitude of writers on the mind cure; indeed, he is the only one who seems to have taken a clearly philosophic view of the subject, and who has given an explanation of the so-called mind cure phenomena which can be read without exciting the antagonism of nine-tenths of his readers. His design in the book is to "illustrate the correspondence of the soul and body, their mutual action and reaction, and to demonstrate the casual relation of disorded mental states to diseased physiological action." Unlike the metaphysicians, Dr. Evans believes in something more than mere mental operations in the treatment of disease. He advocates message, the Swedish movement cure, and magnetic treatment. It is asserted by the apostles of some of the mind-cure schools that faith is an unnecessary element in the treatment of patients, and that there is really no such thing as disease. Dr. Evans does not agree with either of these statements of belief. He recognizes the existence of disease, and says, "There are two things in a patient necessary to the reception of a spiritual sanative influence. One is a desire to get well. The other is a faith in the efficiency of the remedial agency. Without these two the cure of disease by any mode of treatment is, to say the least, if not impossible, exceedingly difficult." And unless the patient has faith in the physician and mode of treatment, he adds, "the case may be dismissed or treated with pure water-drops, or cracker pills, or homeopathic pellets. The only sure thing about the case will be the entry of the fee upon the physician's book or into his pocket." Although we are not inclined to accept all the theories of Dr. Evans, we cordially commend it to those readers who are interested in the subject as well worthy reading and consideration.

